# CMA: how long does it last for?

**DOI:** 10.1186/2045-7022-1-S1-S51

**Published:** 2011-08-12

**Authors:** Nikolaos Papadopoulos

**Affiliations:** 1 University of Athens, Athens, Greece

## 

The natural history of cow’s milk allergy is usually benign. Among non-IgE mediated forms, proctocolitis resolves in practically all cases close to the first year of life; FPIES is dealt with more conservatively, nevertheless, it also resolves in most cases before the age of 5 years. All studies focusing on IgE mediated CMA have demonstrated a time-dependent decrease of prevalence due to the development of tolerance in increasing proportions of the relevant populations. Epidemiological studies have also been ubiquitous in confirming that, in contrast to childhood, cases of IgE-CMA in adults are very rare. Although it is not clear whether such cases have a childhood onset, there is no doubt that only a handful of pediatric patients will continue to suffer post adolescence. Nevertheless, it has been put forward that CMA may currently last longer than previously suggested, with proportions developing tolerance at school age dropping from ~90% to ~40% in the two most cited studies. In order to inform such discussion, factors that may affect the natural history should be taken into account. These include, but are not confined to, severity of the disease, pattern of IgE sensitization and events relevant to antigen exposure. The infant diet may be crucial, however, few studies have addressed the effects of different variations of it on natural history. A considerable proportion of CMA children may tolerate partially hydrolyzed and/or heated milk, however the effects of these on the natural history are not clear. Finally, several successful attempts to introduce CM in the context of SOTI protocols, seems to be able to permit milk consumption in these children, while inducing tolerance in some.

